# Supplementary material for: Multiple Levels of Triggered Factors and the Obligated Requirement of Cell-to-Cell Movement in the Mutation Repair of Cucumber Mosaic Virus with Defects in the tRNA-like Structure
Source: Biology (Basel). 2022 Jul 13;11(7):1051. doi: 10.3390/biology11071051 (PMC9312275; doi:10.3390/biology11071051)
Supplement: Supplementary file 1 [file biology-11-01051-s001.zip › biology-1792064-supplementary/biology-1792064 supplementary WB.pptx]

## Slide 1
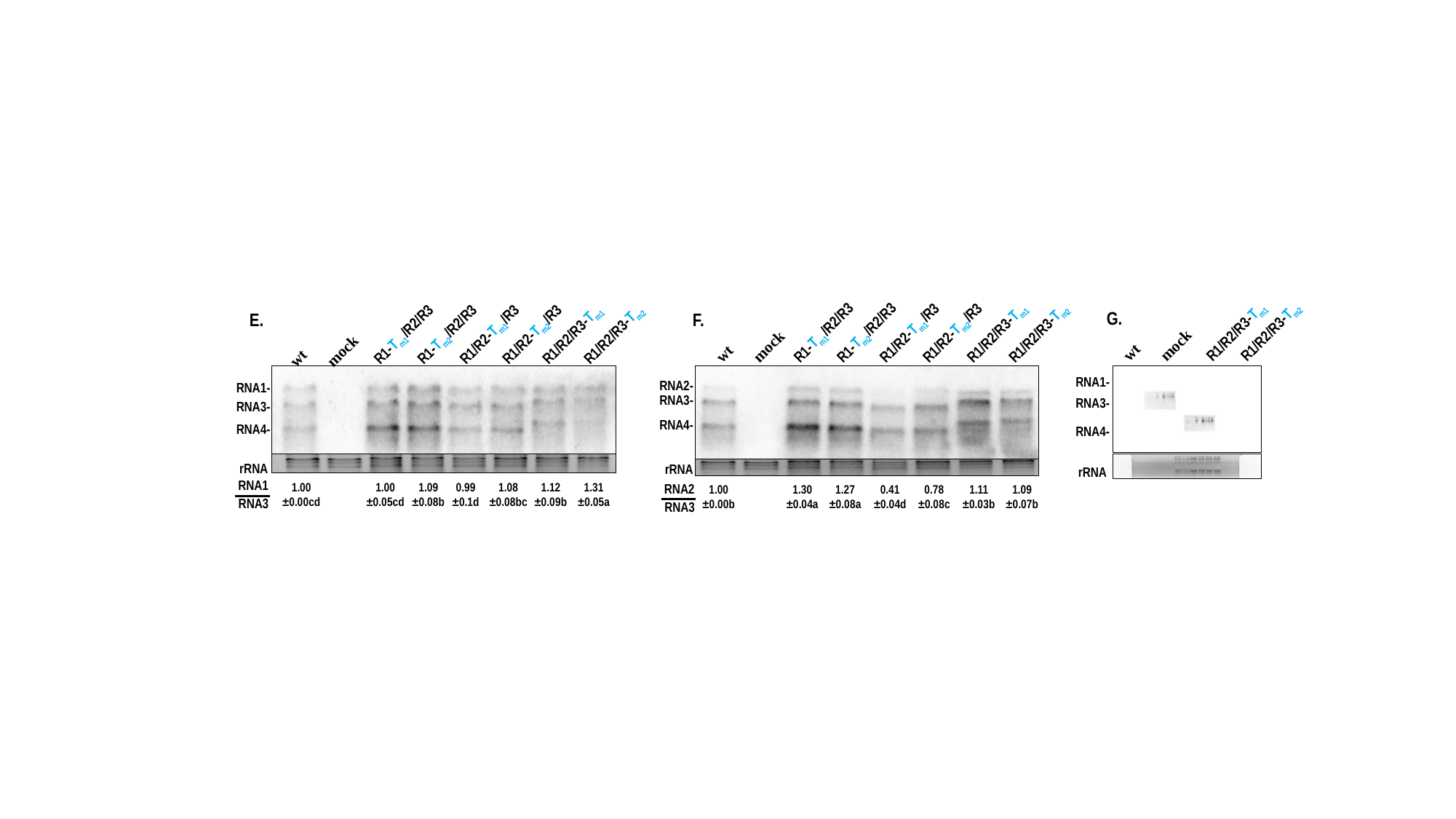

G.
R1/R2/R3-Tm1
R1/R2/R3-Tm2
wt
mock
RNA1-
RNA3-
RNA4-
rRNA
E.
F.
R1-Tm1/R2/R3
R1-Tm2/R2/R3
R1/R2-Tm1/R3
R1/R2-Tm2/R3
R1/R2/R3-Tm1
R1/R2/R3-Tm2
R1-Tm2/R2/R3
R1/R2-Tm1/R3
R1-Tm1/R2/R3
R1/R2/R3-Tm1
R1/R2/R3-Tm2
R1/R2-Tm2/R3
wt
mock
wt
mock
RNA2-
RNA1-
RNA3-
RNA3-
RNA4-
RNA4-
 rRNA
rRNA
RNA1
1.00
±0.00cd
1.00
±0.05cd
1.09
±0.08b
0.99
±0.1d
1.08
±0.08bc
1.12
±0.09b
1.31
±0.05a
RNA2
1.00
±0.00b
1.30
±0.04a
1.27
±0.08a
0.78
±0.08c
1.11
±0.03b
1.09
±0.07b
0.41
±0.04d
RNA3
RNA3

## Slide 2
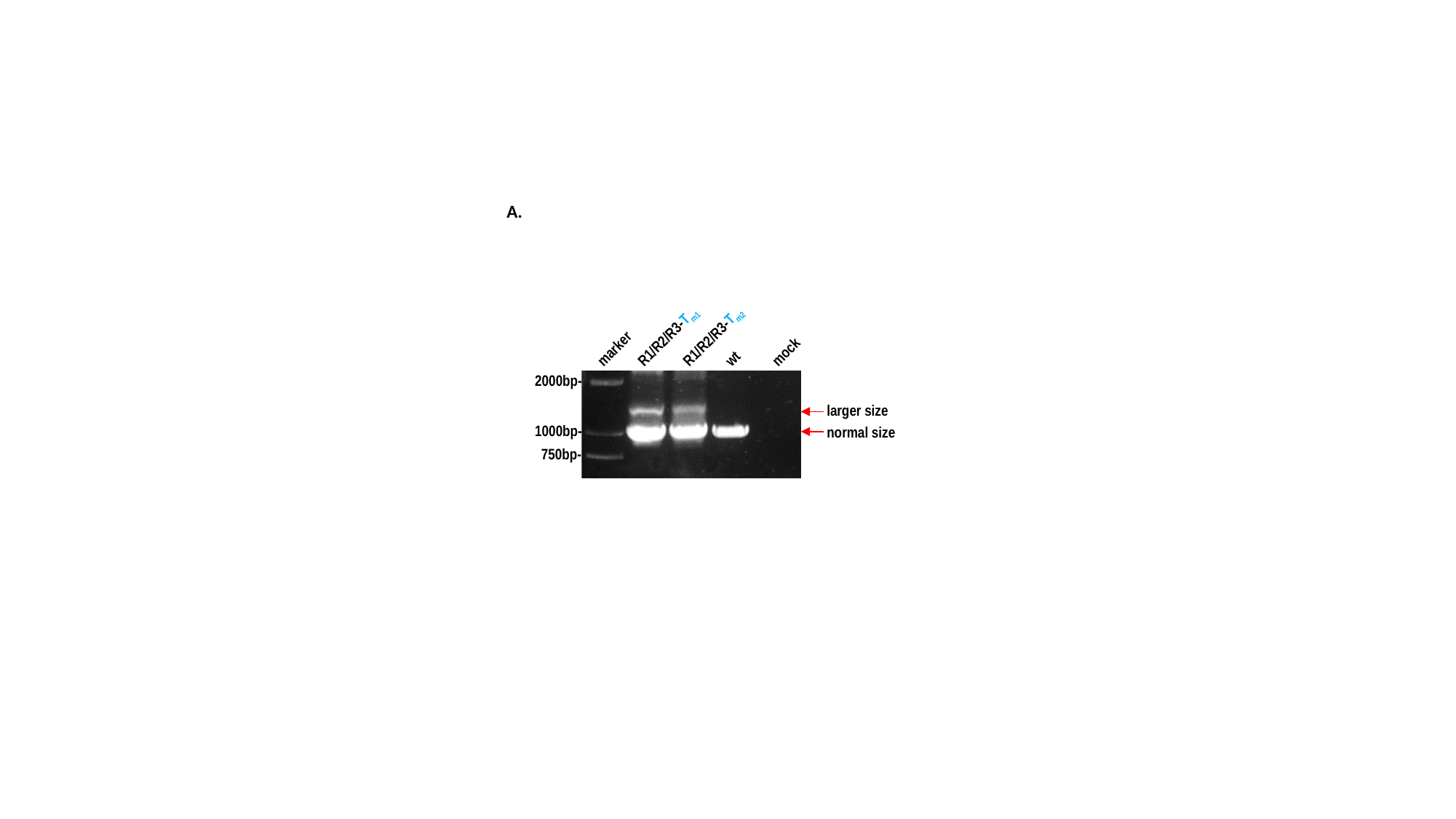

A.
R1/R2/R3-Tm1
R1/R2/R3-Tm2
wt
mock
marker
2000bp-
larger size
1000bp-
normal size
750bp-

## Slide 3
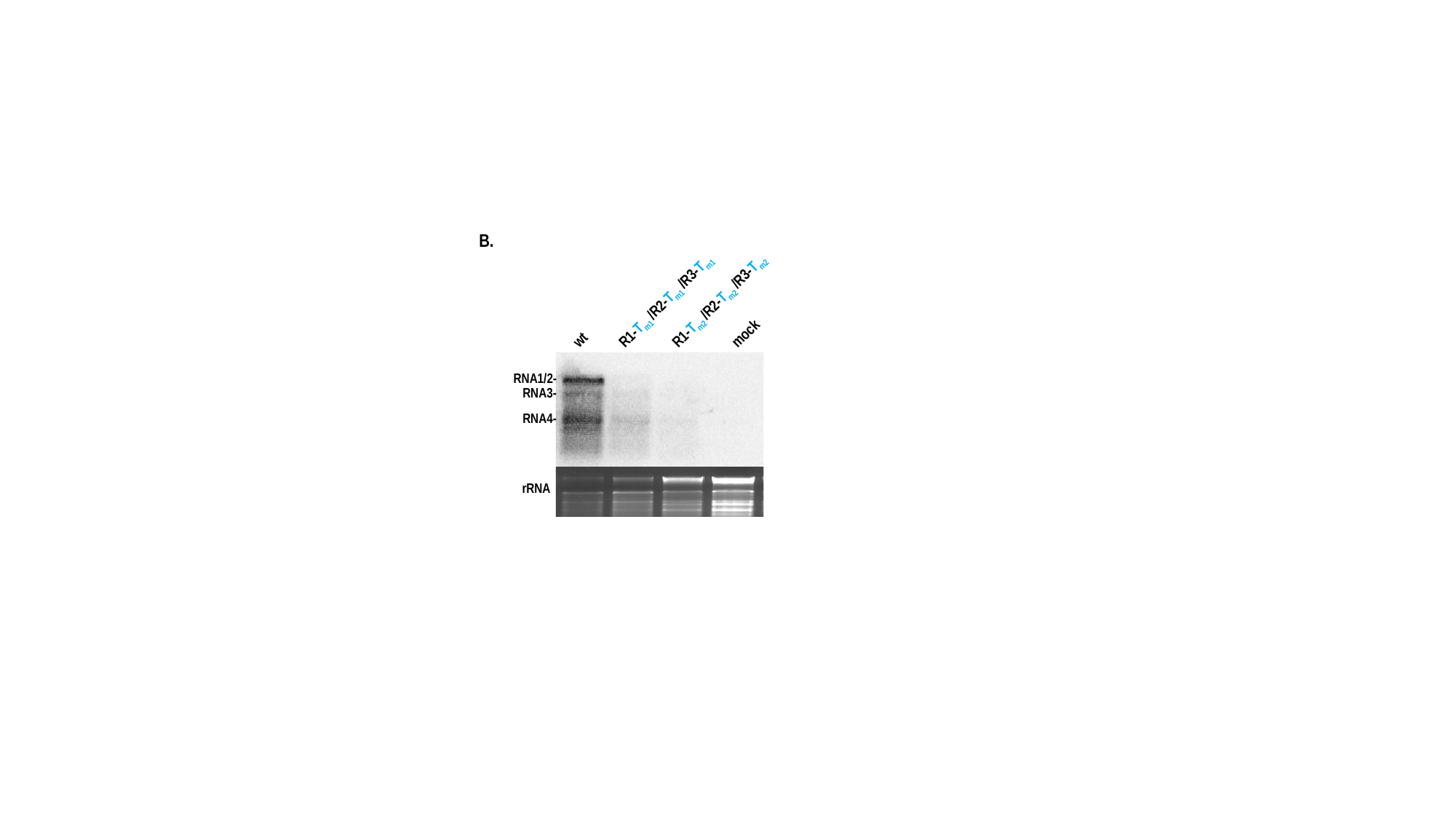

B.
R1-Tm1 /R2-Tm1 /R3-Tm1
R1-Tm2 /R2-Tm2 /R3-Tm2
wt
mock
RNA1/2-
RNA3-
RNA4-
rRNA

## Slide 4
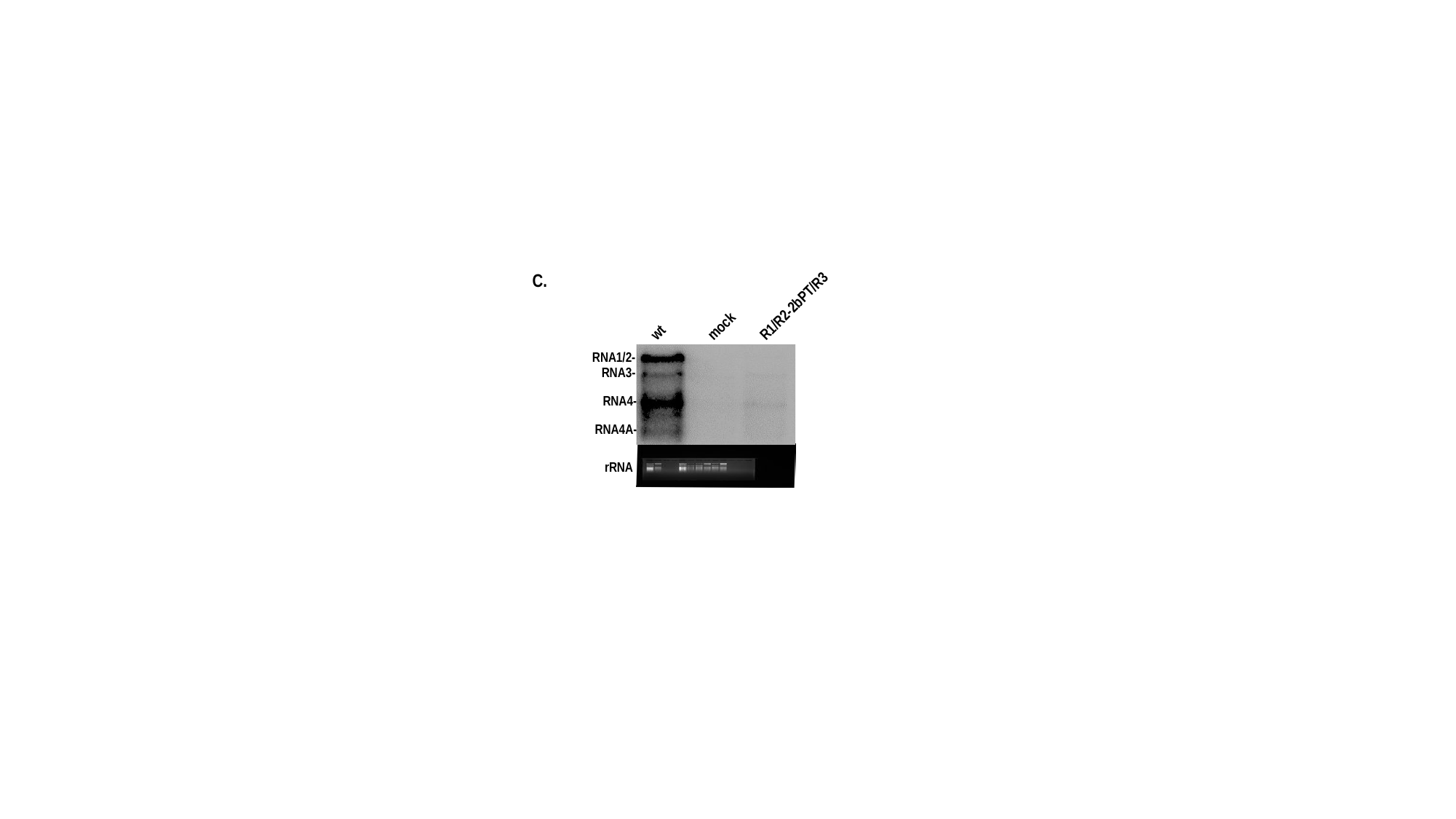

C.
R1/R2-2bPT/R3
wt
mock
RNA1/2-
RNA3-
RNA4-
RNA4A-
rRNA

## Slide 5
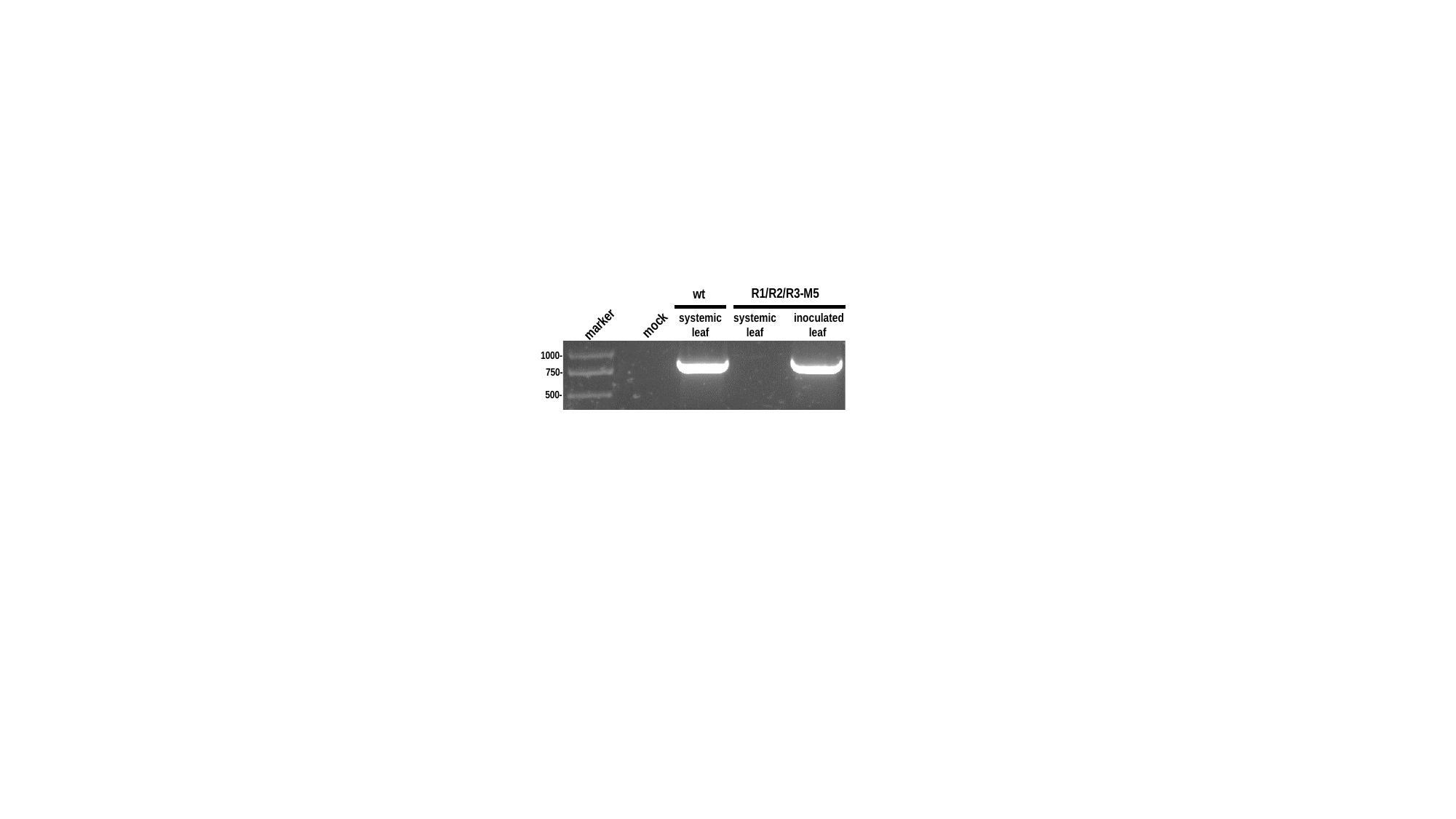

R1/R2/R3-M5
wt
mock
systemic leaf
systemic leaf
inoculated leaf
marker
1000-
750-
500-

## Slide 6
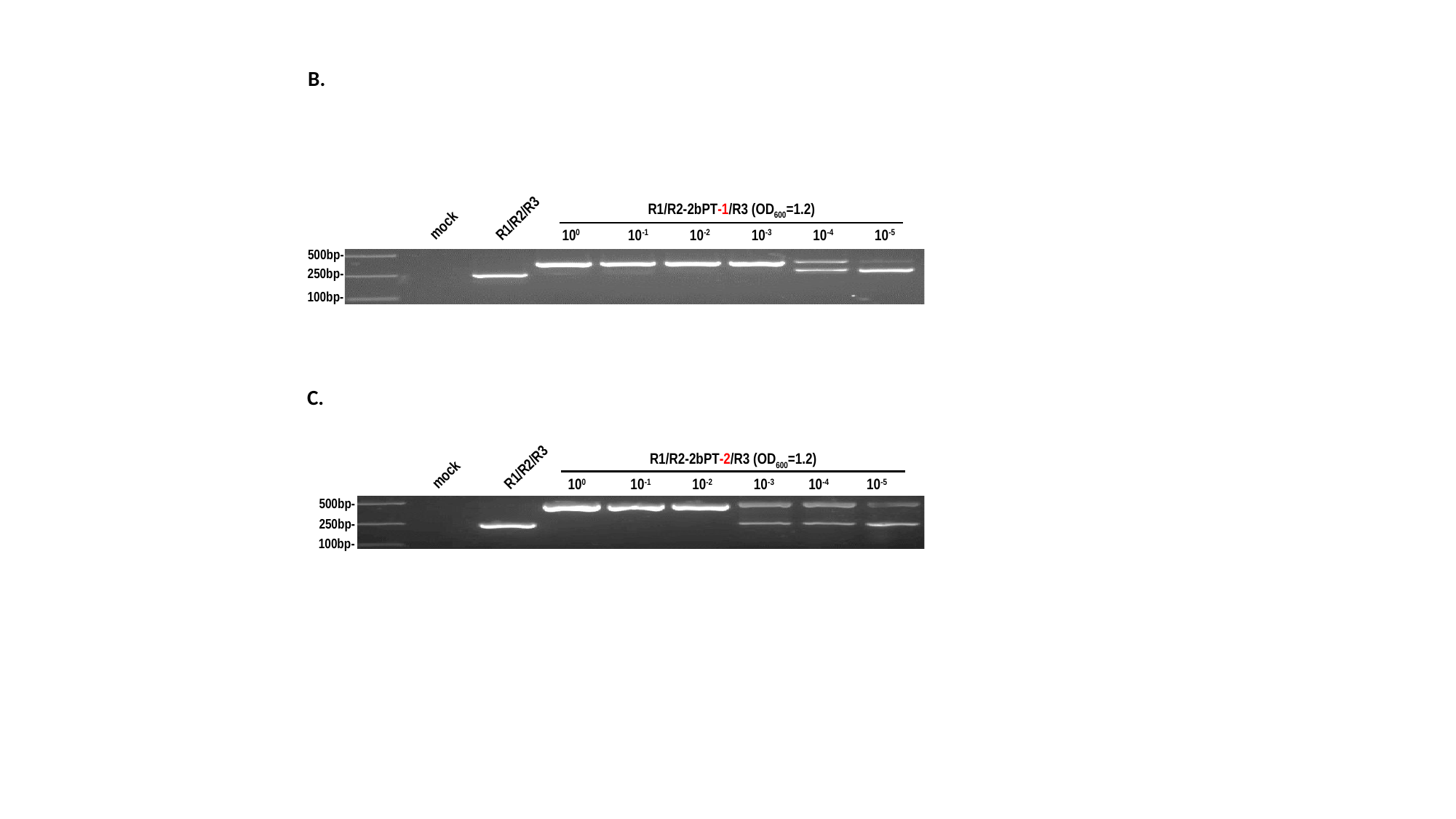

B.
R1/R2-2bPT-1/R3 (OD600=1.2)
R1/R2/R3
mock
100 10-1 10-2 10-3 10-4 10-5
500bp-
250bp-
100bp-
C.
R1/R2-2bPT-2/R3 (OD600=1.2)
R1/R2/R3
mock
 100 10-1 10-2 10-3 10-4 10-5
500bp-
250bp-
100bp-
