# Supplementary material for: Multiple Levels of Triggered Factors and the Obligated Requirement of Cell-to-Cell Movement in the Mutation Repair of Cucumber Mosaic Virus with Defects in the tRNA-like Structure
Source: Biology (Basel). 2022 Jul 13;11(7):1051. doi: 10.3390/biology11071051 (PMC9312275; doi:10.3390/biology11071051)
Supplement: Supplementary file 1 [file biology-11-01051-s001.zip › biology-1792064-supplementary/biology-1792064-supplementary Figures and tables.pdf]

Table S1. Plasmids used in this study.

Mutants	Original sequences	Mutant sequences
pR1-T_{m1}	CTAGC^{3294}ACAACGTGCTAG	CTAGC^{3294}A**GGATCCACTAGTCCCGGG**TGCTAG
pR1-T_{m2}	CTAGC^{3294}ACAACGTGCTAG	CTAGC^{3294}A**GGATCCACTAGTCCCGGG**CAACGTGCTAG
pR2-T_{m1}	CTAGC^{2988}ACAACGTGCTAG	CTAGC^{2988}A**GGATCCACTAGTCCCGGG**TGCTAG
pR2-T_{m2}	CTAGC^{2988}ACAACGTGCTAG	CTAGC^{2988}A**GGATCCACTAGTCCCGGG**CAACGTGCTAG
pR3-T_{m1}	CTAGC^{2152}ACAACGTGCTAG	CTAGC^{2152}A**GGATCCACTAGTCCCGGG**TGCTAG
pR3-T_{m2}	CTAGC^{2152}ACAACGTGCTAG	CTAGC^{2152}A**GGATCCACTAGTCCCGGG**CAACGTGCTAG
pR2-2bPT	TCTGA^{2661}GGCCTCT	TCTGA^{2661}G**TAATAGGGATCCACTAGTCCCGGG**GCCTCT
pR3-M5	CCGAC^{548}GTATGATTGTCCTAT	CCGAC^{548}GgcTGcaTGcCCTAT
pR2-2bPT-1	TCTGA^{2661}GGCCTCT	TCTGA^{2661}G**TAATAGGGATCC-100 bp fragment from TVBMV-CCCGGG**GCCTCT
pR2-2bPT-2	TCTGA^{2661}GGCCTCT	TCTGA^{2661}G**TAATAGGGATCC-200 bp fragment from TVBMV-CCCGGG**GCCTCT

Remarks: The inserted bases were bold, and mutated sites were indicated by low case.

Table S2. Primers used in this study.

DNA Oligos	Sequence (5'-3')	Purpose
R1-T_{m1}-F	**TC**GGATCCACTAGTCCCGGGTGCTAGTTTCAGGGTACGGGTGC	Construction of R1-T_{m1}
R1-T_{m1}-R	**AT**GGATCCTGCTAGAAGTACACGGACCGAAG	
R1-T_{m2}-F	**TC**GGATCCACTAGTCCCGGGCAACGTGCTAGTTTCAGGGTACGGGTG	Construction of R1-T_{m2}
R1-T_{m2}-R	**AT**GGATCCTGCTAGAAGTACACGGACCGAAG	
R2-T_{m1}-F	**TC**GGATCCACTAGTCCCGGGTGCTAGTTTCAGGGTACGGGTGC	Construction of R2-T_{m1}
R2-T_{m1}-R	**AT**GGATCCTGCTAGAAGTACACGGACCGAAG	
R2-T_{m2}-F	**TC**GGATCCACTAGTCCCGGGCAACGTGCTAGTTTCAGGGTACGGGTG	Construction of R2-T_{m2}
R2-T_{m2}-R	**AT**GGATCCTGCTAGAAGTACACGGACCGAAG	
R3-T_{m1}-F	**TC**GGATCCACTAGTCCCGGGTGCTAGTTTCAGGGTACGGGTGC	Construction of R3-T_{m1}
R3-T_{m1}-R	**AT**GGATCCTGCTAGAGGTACACGGACCGAAG	
R3-T_{m2}-F	**TC**GGATCCACTAGTCCCGGGCAACGTGCTAGTTTCAGGGTACGGGTG	Construction of R3-T_{m2}
R3-T_{m2}-R	**AT**GGATCCTGCTAGAGGTACACGGACCGAAG	
R2-2bPT-F	**TC**GGATCCACTAGTCCCGGGGCCTCTCGTTTAGAGTTATCGGCG	Construction of R2-2bPT
R2-2bPT-R	**AT**GGATCC*CTATTA*CTCAGACTCGGGTAACTCCGCC	
TVBMV-7600-F	**AT**GGATCCCAGCACCTCTGGATACTCTACTAG	Construction of R2-2bPT-1 and R2-2bPT-2
TVBMV-7699-R	**AT**CCCGGGATCCCGACACTCCATGGTC	
TVBMV-7799-R	**AT**CCCGGGTGTCAATGAGCTGTCAAAACGACT	
R3-M5-F	CAACCGACGGCTGCATGCCCTATGGAA	Construction of R3-M5
R3-M5-R	TTCCATAGGGCATGCAGCCGTCGGTTG	
Fny3-pCB301-F	AGTTCATTTCATTTGGAGAGGGTAATCTTACCACTGTGTGTGTGC	
Fny3-pCB301-R	GTGGAGATGCCATGCCGACCCTGGTCTCCTTTTGGAGGCC	
pCB301-800-F	GGGTCGGCATGGCATCTCCA	
pCB301-767-R	CCTCTCCAAATGAAATGAACTTCCTTATATAGAGGA	
R1-2658-F	ATGGCTACGAAGGCCCTTC	Detection of TLS mutants in RNA1
R2-2510-F	AGAATCGACGGGAACGAGGT	Detection of TLS and 2b mutants in RNA2
R2-2760-R	GGGGAGGTTTCAGAAAGCACC	Detection of 2b mutants in RNA2
R3-1385-F	CCAACTATTAACCACCCAACCTTTGT	Detection of TLS mutants in RNA3
R1-R	TGGTCTCCTTTTAGAGACCCCCACG	Detection of TLS mutants in RNA1
R2/3-R	TGGTCTCCTTTTGGAGGCCCC	Detection of TLS mutants in RNA2/3
R3-1883-F	GTTATTGTCTACTGACTATATAGAGAGTGTT	Clone of the 3'half of larger RNA3
R3-198-F	AAATGGCTACTGAGTGTGACC	Detection of 3a mutation in RNA3
R3-1040-R	GCATCGCGTCACAGATGTCTAC	
R1-NT-F	TGTGACCTGTTTAAACAGTTTTCTTATTG	RNA1 probe for northern blot
R1-NT-R	ACACAATGTGTTTAGTGACTTCAGAC	

R2-NT-F	AGAGCGCGTTCAAATCTCAGA	RNA2 probe for northern blot
R2-NT-R	TTCGTTACCGGCGAACCAAT	
R3-NT-F	GTACTGGTTTATCAGTATGCCGC	RNA3 and RNA4 probe for northern blot
R3-NT-R	GACTGGGAGCACTCCAGAT	
CM-NT-F	AAACTGTCTGAAGTCACTAAAC	CMV genome and subgenome probe for northern blot
CM-NT-R	TGGTCTCCTTTTGGAGGC	

Remarks: Protective bases were displayed in a bold font. Endonuclease sites was displayed by underline. Stop codons was displayed in italics.

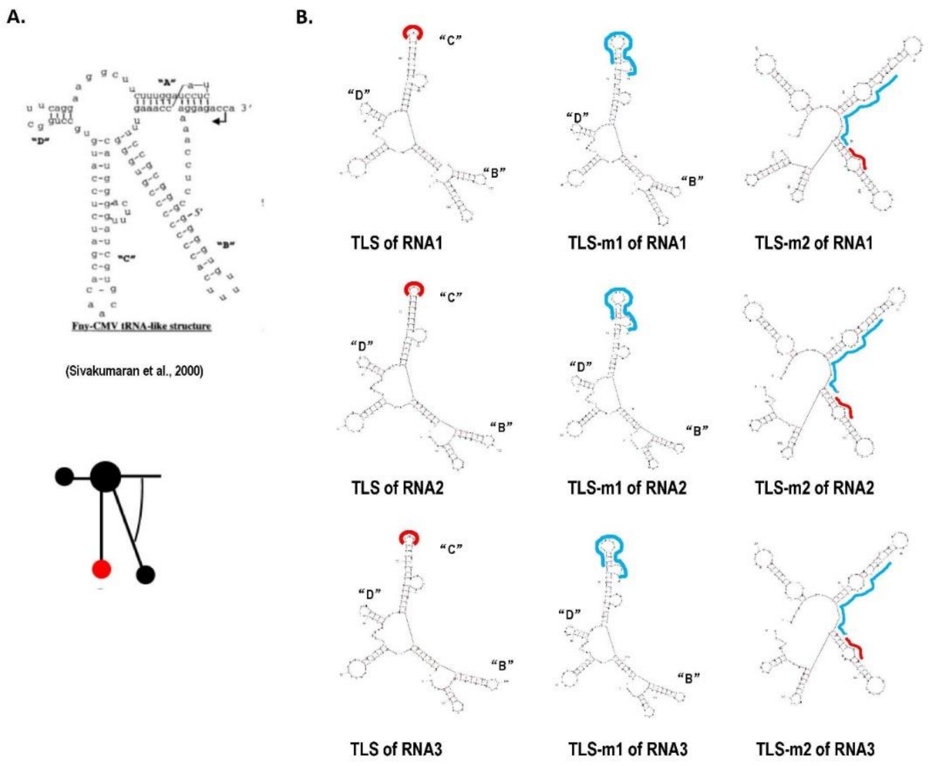

Figure S1. Alignment of tRNA-like structure (TLS) region among wt RNAs, T_{m1} and T_{m2} mutants of cucumber mosaic virus.Secondary structure of TLS in CMV and corresponding schematic representations. caacg loop was indicated by red color. B. Alignment of TLS among wt RNAs, T_{m1} and T_{m2} mutants of CMV. caacg loop sequences were indicated by opened red circles in wt RNAs and red lines in T_{m2} mutants. 18 nt new sequences were indicated by blue lines.

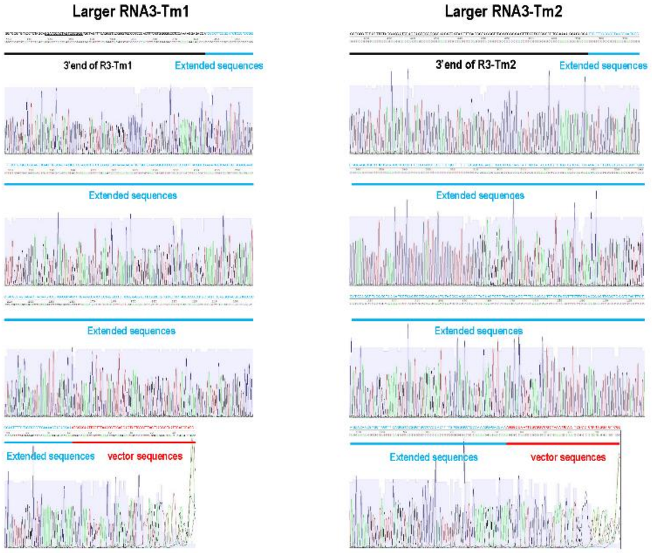

Figure S2. Sequencing results of RT-PCR clones: Red nucleotides indicate 18nt insertion sequences in R3-T_{m1} and R3-T_{m2}. Blue nucleotides indicate the extended sequences. Purple nucleotides indicate vector sequences.

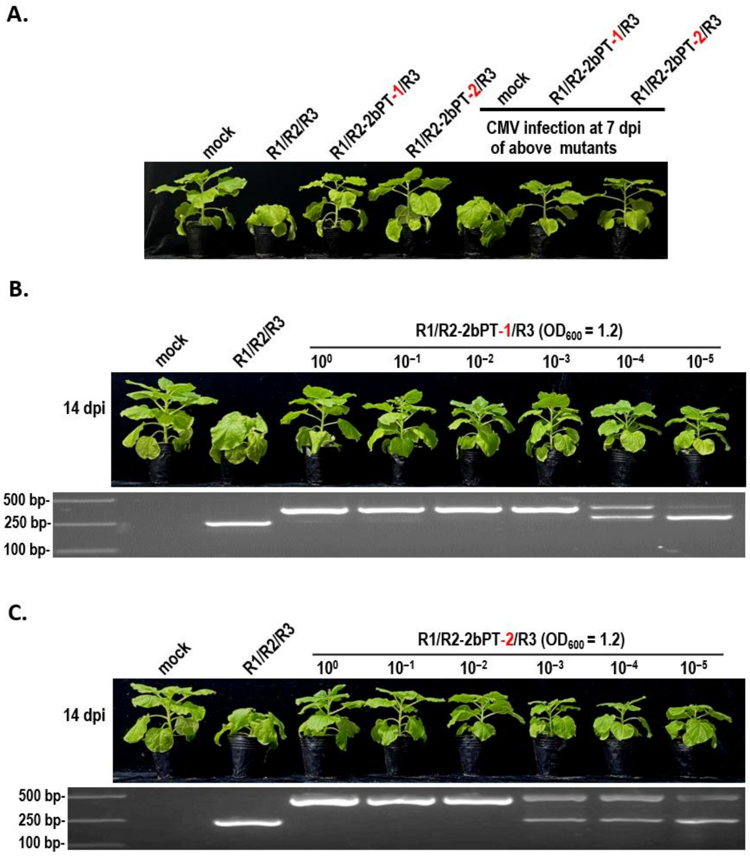

Figure S3. Dilution inoculation of attenuated vaccines of CMV and mutant repair assay. (A) Mutants cross protection assay of CMV_{Fny}. R1, RNA1 of CMV_{Fny}; R2, RNA2 of CMV_{Fny}; R3, RNA3 of CMV_{Fny}. (B) Mutation repair assay under dilution inoculation of R1/R2-2bPT-1/R3. The PCR products showed a band of 350 bp indicating all mutants were existent; the PCR products showed two bands of 250 bp and 350 bp indicating part of the mutants were repaired to wild-type RNA2. (C) Mutation repair assay under dilution inoculation of R1/R2-2bPT-2/R3. The PCR products showed a band of 450 bp indicating all mutants were existent; the PCR products showed two bands of 250 bp and 450 bp indicating part of the mutants were repaired to wild-type RNA2.
